# Stainless Steel Foil-Based Label-Free Modular Thin-Film Electrochemical Detector for Solvent Identification

**DOI:** 10.3390/mi13122256

**Published:** 2022-12-19

**Authors:** Martin Rozman, Miha Lukšič

**Affiliations:** 1 FunGlass—Centre for Functional and Surface Functionalized Glass, Alexander Dubček University of Trenčín, Študentská 2, SK-91150 Trenčín, Slovakia; 2 Faculty of Chemistry and Chemical Technology, University of Ljubljana , Večna pot 113, SI-1000 Ljubljana, Slovenia

**Keywords:** electrochemical sensor, impedance spectroscopy, organic solvents, stainless steel

## Abstract

Most organic solvents are colorless liquids, usually stored in sealed containers. In many cases, their identification depends on the appropriate description on the container to prevent mishandling or mixing with other materials. Although modern laboratories rely heavily on identification technologies, such as digitized inventories and spectroscopic methods (e.g., NMR or FTIR), there may be situations where these cannot be used due to technical failure, lack of equipment, or time. An example of a portable and cost-effective solution to this problem is an electrochemical sensor. However, these are often limited to electrochemical impedance spectroscopy (EIS) or voltammetry methods. To address this problem, we present a novel modular electrochemical sensor for solvent identification that can be used with either an EIS-enabled potentiostat/galvanostat or a simple multimeter. A novel method of fabricating and using a sensor consisting of a thin-film coating of an organic substance on a stainless-steel electrode substrate is presented. The differences in the solubility of the thin film in different solvents are used to distinguish between common organic solvents such as water, ethanol, and tetrahydrofuran.

## 1. Introduction

The development of chemical sensors has a century-long tradition and they have found wide applications in various fields including analytical chemistry, the determination of pathogens and toxins in medicine, forensics, and even industrial quality control [1,2,3,4]. Modern sensor technology is focused on lightweight and portable devices, ease of use, and low operating costs. Some chemical sensors have been widely used outside of specialized professions and can be purchased as commercial products, such as pH-dye litmus paper [5], blood sugar monitors [6], and pregnancy tests [7]. One of the sensor systems that is likely to remain most important in this decade is the COVID-19 rapid antigen test [8,9]. The main reason such sensors have become so popular is that they are cheap, disposable, and easy to use. Since most of these sensors are designed to be colorimetric, efforts have been made to modify them to respond to an electrical signal that would allow easily digitized measurements. This is especially important where large numbers of samples are involved or where the results need to be sent immediately to multiple recipients [10,11]. For this reason, electrochemical biosensors are very attractive because the size of the biosensing unit itself is, in most cases, similar to or even smaller than conventional chemical sensors. However, they often require potentiostat/galvanostat instruments or frequency response analyzers (FRA) [12], which are considerably more expensive than disposable sensor units. One of the approaches to reducing costs has been to develop specialized electrochemical instruments with a more limited measuring range. Such examples include electrochemical biosensors for glucose and pregnancy tests, which result in much lower instrument unit costs to the point that the instrument unit itself can be considered disposable. Nevertheless, the manufacturing costs of the instrument unit are typically ten or more times those of the sensing element itself so it is treated as a non-disposable component in many industries.

The chemical industry relies heavily on various methods to determine the composition and quality of the components it uses to transform raw materials into desired products. Since the quality control of raw materials such as powders and liquids is of great importance to the development of any product, manufacturers spare no expense on instruments that have higher acquisition and operating costs such as chromatography, X-ray fluorescence, and nuclear magnetic resonance, among others [13,14]. One of the most important areas in the chemical industry is quality control and the study of solvents [15,16] because they are often used in synthesis or maintenance operations such as purification or other types of reconditioning. Because many solvents and solutions are colorless liquids, situations can sometimes arise in which they can be misidentified. Although small volumes may not be a problem in the laboratory, misidentification can occur with canisters or other large-volume containers because of the potential for chemical mix-ups and possible side reactions that can lead to the release of toxic gases or even explosions [17,18]. Numerous incidents have been reported due to hasty actions and refilling the wrong container, resulting in several casualties [19]. For this reason, health and safety authorities often call for the installation of safety systems that can prevent such disasters [20]. One such option is the installation of electrochemical or spectroscopic detectors that automatically lock valves and prevent the mixing of different chemicals. The electrochemical detectors used for such tasks often rely on impedance measurements, such as electrochemical impedance spectroscopy (EIS) and open-circuit potential (OCP) [21], because these techniques are usually the most responsive [22,23]. The major drawback of these methods is that they often require expensive potentiostats with an accuracy of less than one millivolt or FRA that are capable of performing impedance measurements [24]. There have been some attempts to assemble detectors that rely on resistance measurements since these can be made with much less expensive multimeters, but most have limited performance and can only distinguish between two different solvents [25] or can only detect solvents in the vapor state [26]. An alternative to these is field emission transistors (FET) [27,28], which can detect multiple organic compounds, but they are much more expensive to manufacture. In this paper, we present a method for building a chemical sensor that uses thin-film-coated stainless-steel sheet electrodes and can be operated with a simple multimeter. The sensor is modular and can discriminate between exposure to multiple solvents such as water, ethanol, and tetrahydrofuran.

## 2. Materials and Methods

### 2.1. Materials

Lithium carbonate (Li${}_{2}$CO${}_{3}$; 99%), potassium chloride (99.999 Suprapur), and acetone (p.a.) were purchased from Merck, whereas ethanol (96%) and tetrahydrofuran (THF; 99.9%) were obtained from Sigma-Aldrich. Water was purified using a Seralpur Pro 90C unit in combination with a USF Elga laboratory unit. The electrodes were constructed from AISI 316 stainless steel (TBJ Industrieteile GmbH, Leipzig, Germany) foil. Ethanol-susceptible thin films were prepared using an alcohol-based ink marker (Lumocolor duo 348 WP4 Permanent marker Red, Staedtler, Nuremberg, Germany ) and THF-susceptible thin films were prepared using epoxy-based composite adhesives (2K Metal Epoxy Adhesive 86490; Kent, Fife, UK). Insulating tape (Tesa, Germany) and adsorption paper (Multi Fun, Paloma, Sladki Vrh, Slovenia) were purchased locally. Euroturbo microscope slides D100001 (Deltalab, Barcelona, Spain) were used as glass substrates and for device encapsulation.

### 2.2. Solution Preparation

An amount of 100 mL of stock solution of electrolyte was prepared by dissolving 0.6969 g of lithium carbonate in demineralized water (electrolyte concentration 0.0943 mol/dm${}^{3}$). Mixtures of Li${}_{2}$CO${}_{3}$ solution with water, ethanol, and THF were prepared at volume ratios of 9:1 by mixing 18 mL of stock electrolyte solution and 2 mL of water, ethanol, or THF.

### 2.3. Thin-Film-Coated Electrode Preparation

Three electrodes measuring 25 mm × 5 mm and one electrode measuring 25 mm × 20 mm were cut out of the stainless-steel foil. All the electrodes were rinsed with acetone to remove the organic contaminants on the surface and air-dried.

Two different thin films (ethanol- and THF-susceptible) were applied to two smaller electrodes, whereas the third smaller electrode served as an uncoated (blank) electrode. In our device, these three electrodes served as the working electrodes. The larger electrode served as a (pseudo) reference/counter electrode. The ethanol-susceptible thin film (EtOH-STF) was prepared by rubbing a modified alcohol-based ink marker over the surface of the stainless-steel electrode. The marker was cut with a scalpel to create a straight coating area of 5 mm × 5 mm. The marker was then pressed firmly onto the first electrode and rubbed three times over the active surface of the electrode, measuring 20 mm × 5 mm. The film was allowed to dry completely for 2 h. To prepare the tetrahydrofuran-susceptible thin film (THF-STF), Part A and Part B of the composite epoxy-based adhesive were first mixed and stirred with a spatula. The mixture was then applied to the electrode via the doctor blade method, and the surface area of the thin film was 20 mm × 5 mm. The film was left for 6 h to solidify completely.

### 2.4. Thin-Film-Coated Glass Substrate Preparation

Ethanol- and THF-susceptible thin films on glass substrates were prepared in a similar manner to the thin films used to prepare the electrodes, except that the coated area was 30 mm × 15 mm and the glass substrates used were measured 76 mm × 26 mm (microscope slides).

### 2.5. Sensor Device Assembly

**Figure 1:**
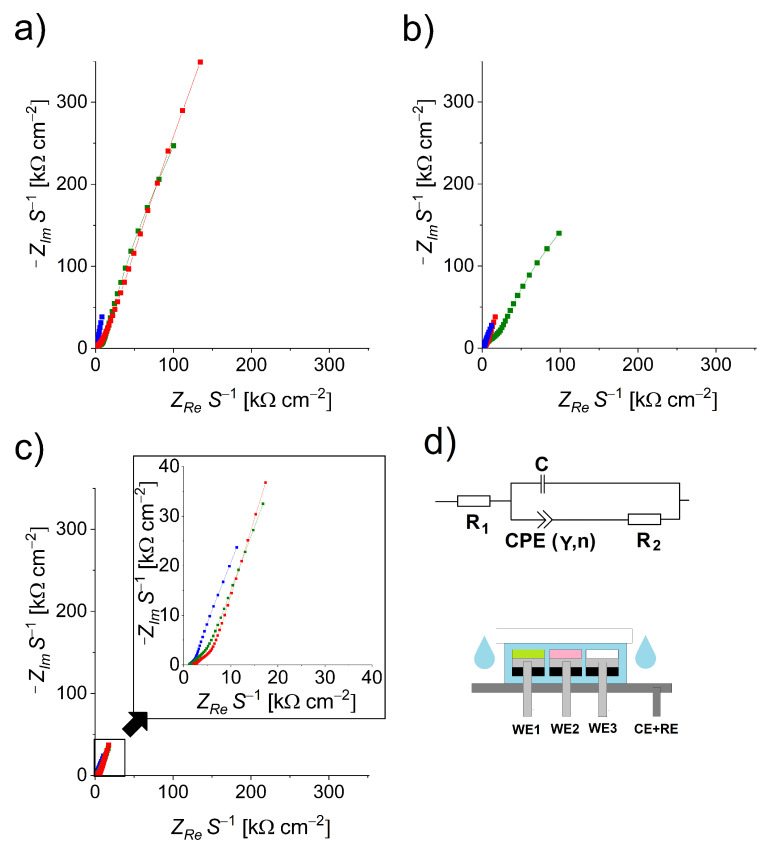
Nyquist plots of the assembled device exposed to different solvents: (**a**) water, (**b**) ethanol, and (**c**) THF, with a magnified plot. Each graph contains measurements for the three working electrodes: uncoated stainless steel (blue), coated with ethanol-susceptible thin film (red), and coated with THF-susceptible thin film (green). (**d**) Equivalent circuit used in fitting the experimental data along with schematics of the assembled device, showing the three separated working electrodes (WE1, WE2, and WE3) and combined reference/counter electrode (RE + CE). The impedances are normalized to the surface area of the working electrodes, *S*.

The insulation tape was first glued to the uncoated side of each of the three smaller electrodes measuring 25 mm × 5 mm. A strip of adsorption paper (kitchen paper towel) measuring 10 mm × 25 mm was then wrapped twice around each of the smaller electrodes, with the paper covering the central exposed surface area. After wrapping the paper, the smaller electrodes were positioned side by side on top of the larger electrode with the exposed area facing away from the electrode and secured to the electrode with two pieces of electrical tape, each measuring 5 mm × 25 mm. Finally, the electrodes were sandwiched between two glass slides (clamped with office paper clips) to secure the position of the electrodes. A schematic representation of the device with the three working electrodes and a combined reference/counter electrode is shown in Figure 1d.

### 2.6. Electrochemical Measurements

Electrochemical impedance spectroscopy (EIS) was performed using the PalmSens4 instrument (PalmSens, Houten, The Netherlands). An amplitude of 10 mV against a fixed potential of 0 V was used [29,30] and scans were performed in a frequency range from 500 kHz to 1 Hz with 9.8 points per decade (57 points in total per measurement). For all measurements, the working electrode (WE) terminal was connected to one of the smaller electrodes (the electrode under study), whereas the counter electrode (CE) and the reference electrode (RE) terminals were combined and connected to the larger electrode (*cf*. Figure 1d).

The cell resistance measurements (CRMs) were performed using a PeakTech 2035 multimeter (PeakTech, Ahrensburg, Germany). The multimeter was set to record the changes in resistance at 10 measurements per second, with each measurement lasting 10 s. Similar to the EIS measurement, the WE terminal was connected to the smaller electrode under study, whereas the CE terminal was connected to the large electrode.

The same measurement protocol was used for both the EIS and CRM measurements: The assembled device was first soaked with stock electrolyte solution (0.0943 mol/dm${}^{3}$ Li${}_{2}$CO${}_{3}$), with the first measurement performed in combination with each of the three WE. The device was then rinsed with ethanol, which remained on the device for 10 s. The device was again rinsed with the electrolyte solution and the second series of measurements was performed separately for each WE. Following the second series of measurements, the device was rinsed with THF, which remained on the device for 10 s. Finally, the THF was removed by rinsing the device with electrolyte solution and the third series of measurements was performed separately for each WE.

### 2.7. Conductometry

The electrical conductivities of the solutions were determined using the InLab 730 conductivity probe (Mettler Toledo, Greifensee, Switzerland) in conjunction with the SevenEasy conductivity meter (Mettler Toledo, Switzerland). Calibration was performed using a 0.02 mol/L aqueous potassium chloride solution. All solutions were thermostatted to 25 °C using the Julabo MP-5 thermostat (Julabo GmbH, Seelbach, Germany). Approximately 20 mL of the solution was required for each measurement.

### 2.8. UV-Vis Transmittance Spectroscopy

For spectrophotometric analysis of thin-film dissolution, thin-film samples were prepared on glass substrates (Section 2.4). The coated glass substrates were first placed in the Varian Cary 50 UV-Vis spectrophotometer (Agilent Technologies, Santa Clara, CA, USA) and their transmittance values were recorded at a 550 nm wavelength. Then, using a dropper, the solvent (ethanol or THF) was applied to the thin films and left to act on them for 30 s. Then, the solvent was dried in air for 15 min. The solvent was then washed off with electrolyte. Finally, the glass substrates with the exposed thin films were placed in the spectrophotometer and the second transmittance reading was recorded.

## 3. Results

The results are presented in two parts. The first part summarizes the electrochemical investigation methods (EIS and CRM) and the second part summarizes the physical properties of the electrodes and solutions (conductivity, spectrophotometry).

### 3.1. Electrochemical Impedance Spectroscopy (EIS)

**Figure A1:**
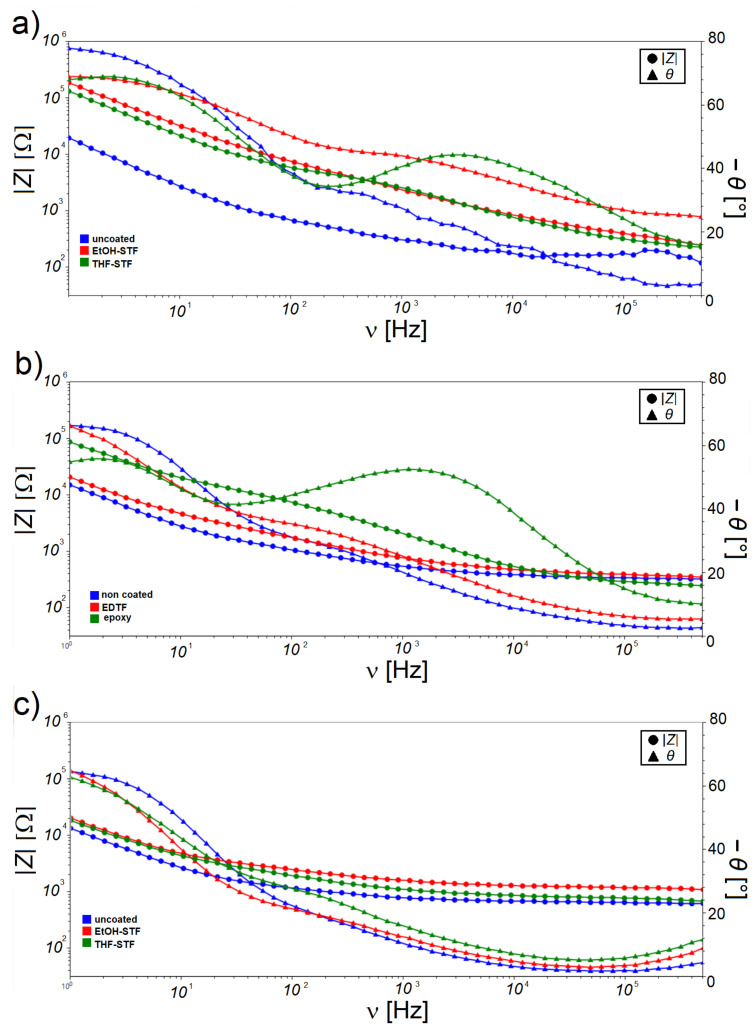
Bode plots of the assembled device exposed to different solvents: (**a**) water, (**b**) ethanol, and (**c**) THF. Each graph contains the measurements for the three working electrodes: uncoated stainless steel (blue), coated with ethanol-susceptible thin film (red), and coated with THF-susceptible thin film (green).

**Figure A2:**
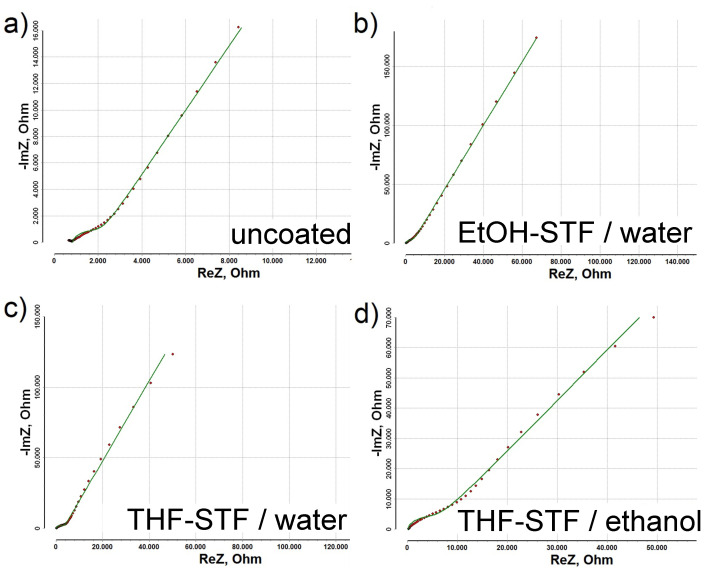
Equivalent circuit fit (lines; *cf.* Figure 1d) of the experimental EIS spectra (points) for (**a**) uncoated WE exposed to water, (**b**) EtOH-STF-coated WE exposed to water, (**c**) THF-STF-coated WE exposed to water, and (**d**) THF-STF-coated WE exposed to ethanol.

EIS was used to study the changes in the working electrode surface of the sensor device assembly when exposed to water, ethanol, and THF. The corresponding Nyquist plots are shown in Figure 1 (information in the form of Bode plots is provided in Appendix A.1 in Figure A1). Figure 1a shows the results of treating the sensor device first with water. We found that both the coated thin-film electrodes had a much higher capacitance and resistance compared to the uncoated electrode. Although the uncoated WE showed a total impedance of about 20 kΩ, the ethanol-susceptible thin-film WE showed a total impedance of almost 190 kΩ, whereas the THF-susceptible thin-film WE showed an impedance of about 130 kΩ. The response of the sensor after exposure to ethanol is shown in Figure 1b: The uncoated WE and the EtOH-STF-coated WE showed similar responses (total impedance of about 20 kΩ), indicating that the thin film dissolved completely. The THF-STF-coated WE showed lower overall impedance compared to the water treatment, but it was still significantly higher (peak value of about 85 kΩ at a frequency of 1 Hz) than in the case of the ethanol-susceptible thin-film case. The last series of measurements was performed after the device was exposed to THF. In Figure 1c, we see that all WE had a similar overall impedance of about 20 kΩ, indicating that THF dissolved both EtOH-STF and THF-STF. The spectra were fitted using a modified equivalent circuit [29], which is shown in Figure 1d. The numerical values of the parameters are given in Table A1 and the fits are shown in Figure A2 in Appendix A.2. The spectra were fitted using an EIS Spectrum Analyzer [31]. In the electrical equivalent circuit shown in Figure 1d, $R_{1}$ covers both the ohmic resistance of the electrolyte and the working electrode, whereas $R_{2}$ represents the polarization resistance (stainless-steel electrode surface and its dimensions). The capacitor element *C* takes into account the effect caused by the material of the electrolyte carrier (cellulose kitchen paper in our case). CPE is an abbreviation for the constant phase element (Y is the admittance and n is the phase parameter). In our case, there are two physical explanations for the CPE behavior: the roughness of the electrode and the varying thickness of the thin-film coating. We mainly attribute the CPE to the topological imperfections of the thin film on the electrode surface. For the sensor with the uncoated WE, the fitting data showed that the resistance $R_{1}$, which measured about 180 Ω, can be related to the specific resistance of the electrolyte, and the parameter $R_{2}$, which measured about 800 Ω, can be attributed to the use of paper wrap material, the stainless-steel surface, and the width of the electrode. This can also be observed in the measurements on devices with EtOH-STF and THF-STF, where the $R_{2}$ parameter increased to about 4.4 kΩ for the EtOH-STF-coated WE and 3.4 kΩ for the THF-STF-coated WE. In all cases, the uncoated WE showed no changes regardless of the solvent present, indicating that the electrode material and other components of the device were not sensitive to water, ethanol, or THF.

### 3.2. Cell Resistance Measurement (CRM)

Compared to EIS, the CRM method is a simpler and faster method. For practical reasons, the use of a simple multimeter in conjunction with the sensor device is desirable. CRMs were performed to investigate the possibility of simplifying the electrochemical measurement. The recorded data are shown in Table 1. When the multimeter was connected to the uncoated WE, the active area of the stainless-steel electrode was 0.5 cm${}^{2}$ and the total resistance of the device was 324±5 kΩ. Similar values were also measured in the cases where the uncoated WE was treated with ethanol and THF. The electrodes coated with an ethanol-susceptible film and a THF-susceptible film exhibited much greater total resistance (approximately 6380 and 5340 kΩ for the ethanol- and THF-susceptible films, respectively). Interestingly, the WE with the EtOH-STF coating exhibited higher resistance than the THF-STF-coated WE. This observation is consistent with the EIS measurement, where the EtOH-STF-coated WE had a slightly higher overall impedance. When the ethanol-susceptible thin-film-coated WE was treated with ethanol or THF and the THF-susceptible thin-film-coated WE was treated with THF, the recorded resistance decreased significantly and approached values characteristic of the uncoated electrode. The data obtained with the CRMs were consistent with the EIS measurements, showing that ethanol significantly affected the ethanol-susceptible thin film, whereas the epoxy-based THF-susceptible thin-film-coated WE was less affected. On the other hand, THF completely removed both the ethanol- and THF-susceptible thin films. These measurements show that CRMs can be a suitable detection method in the case of our simple electrochemical sensors.

### 3.3. Conductometry Measurements

The conductivities of the solutions were determined at 25 °C. The concentration of the Li${}_{2}$CO${}_{3}$ stock solution was 0.0943 mol/dm${}^{3}$. Mixtures of the stock solution with water, ethanol, and THF were prepared at volume ratios of 9:1 (18 mL Li${}_{2}$CO${}_{3}$ stock solution and 2 mL water, ethanol, or THF). Data for the specific conductivities are given in Table 2. We can see that the conductivities of the mixtures were lower than the conductivity of the stock electrolyte due to electrolyte dilution. However, the effect of ethanol and THF on lowering the conductivity was greater than that of water. This is because the addition of the organic solvents lowered the dielectric constant of the mixture. The mixture of the electrolyte solution with THF had a slightly lower conductivity than the mixture with ethanol (the dielectric constant of ethanol at 25 °C was about 24.6 and that of THF was 7.6 [32]). The conductometric measurements were consistent with the EIS/CRM results for the sensor devices exposed to various solvents that dissolved the thin-film coating. In these cases, the measured resistance values varied by up to 25%, which was consistent with the changes in the specific electric conductivities of the different solvent mixtures. Although these changes may seem relatively high, it should be noted that the resistance of the unexposed device was up to 20 times higher than the resistance measured after it was exposed to a particular solvent. Thus, the undamaged thin films had considerably higher resistance, and this measurement was less affected by the dilution of the electrolyte, which can introduce errors.

### 3.4. Spectrophotometric Analysis of Thin Films

A spectrophotometric study of the thin films was performed to optically confirm the solubility of the thin films in various solvents. A glass substrate was coated with ethanol- and THF-susceptible thin films (Section 2.4), the sample was placed in the spectrophotometer, and the transmittance was measured at a wavelength of 550 nm. The recorded transmittance data are shown in Table 3. It was observed that exposure to ethanol almost completely removed the EtOH-STF, whereas the THF-STF was only slightly affected, as indicated by a slight change in transmittance. Exposure to THF resulted in the complete dissolution of both thin films, as evidenced by an overall increase in transmittance. The recorded data were, therefore, consistent with the EIS measurements and CRMs, suggesting that the different thin films interacted differently with the solvent with which they were in contact, regardless of the electrodes or other components used to construct the chemical sensor.

## 4. Discussion

Combining the EIS and CRM data showed that it is possible to predict what solvent an electrochemical sensing device has been exposed to by observing the resistance response of the device. The CRM method also had the advantage of being faster compared to EIS and could take continuous measurements. It was also observed that the device did not require mechanical interaction, except for rinsing, where the capillary effect allowed the active surface of the electrode to be wetted with the selected solvent or electrolyte. The device was also able to distinguish between the three types of solvents, with the resistance response at different electrodes clearly indicating which solvent was present: low resistance at one electrode signaled water, two electrodes signaled ethanol, and three electrodes signaled THF. It would be possible to extend the capabilities of the sensor to other organic solvents using different thin films that respond specifically to polar, aprotic, acidic, basic, or redox-active chemicals. From an instrumentation perspective, the CRM method has a particular advantage over EIS in that it can perform measurements on multiple WEs simultaneously. In the case of EIS, simultaneous measurements are more difficult because the induced amplitude of the AC current at different frequencies could interfere with the measurements, which would bias the collected data. Finally, the small size of the sensing device combined with the low-cost CRM instrumentation would be ideal for quality and safety control in large-scale industries, as it could be easily implemented and automated. Thus, the system presented could be used as a valuable fail-safe to prevent chemical mix-ups or as a quick quality control tool in the lab or outdoors. Another benefit of such a device architecture could be to rapidly test a single solvent against multiple coatings. This could be useful for forensic analysis and emergency response teams, as it could quickly determine what protective equipment to use against chemical spills or other industrial accidents.

## Figures and Tables

**Table 1:** Cell resistance measurements per electrode surface area, R/S, of assembled sensor devices using an aqueous lithium carbonate electrolyte. The data show a response to ethanol and THF exposure, as well as the behavior of the non-exposed device. Uncertainties were estimated by repeating the measurements three times. The error of the multimeter used for the CRMs was 0.1%.

Solvent	Working Electrode	R/S [kΩ/cm${}^{2}$]
No exposure	uncoated	628±10
	EtOH-STF	12,760 ±100
	THF-STF	10,680 ±100
Ethanol	uncoated	664±10
	EtOH-STF	736±10
	THF-STF	4442±100
THF	uncoated	656±10
	EtOH-STF	772±10
	THF-STF	790±10

**Table 2:** Specific electrical conductivities of the solutions used in the experiments, determined at 25 °C. Stock electrolyte was a 0.0943 mol/dm${}^{3}$ aqueous solution of Li${}_{2}$CO${}_{3}$, and the mixtures with water, ethanol, and THF were prepared at volume ratios of 9:1. The conductivity of the water used to prepare the solutions was less than 1 μS/cm.

Solvent	Specific Conductivity [mS/cm]
stock electrolyte	11.66
electrolyte-water	10.74
electrolyte-EtOH	7.84
electrolyte-THF	7.68

**Table 3:** Transmittance of thin films on glass substrates recorded at a 550 nm wavelength. The data show a response to ethanol and THF exposure, as well as the behavior of the non-exposed device. Uncertainty was determined from three replicates.

Solvent	Working Electrode (WE)	Transmittance [%]
No exposure	EtOH-STF	1±1
	THF-STF	7±2
Ethanol	EtOH-STF	79±2
	THF-STF	14±5
THF	EtOH-STF	99±1
	THF-STF	96±2

## Data Availability

Data are available from the authors upon reasonable request.

## Abbreviations

The following abbreviations are used in this manuscript:

EtOH	ethanol
THF	tetrahydrofuran
EtOH-STF	ethanol-susceptible thin film
THF-STF	tetrahydrofuran-susceptible thin film
EIS	electrochemical impedance spectroscopy
CRM	cell resistance measurement
WE	working electrode

## Appendix A

### Appendix A.1

EIS data presented in Figure A1 shown in the form of Bode plots.

### Appendix A.2

Values of the equivalent circuit fitting parameters used to model the EIS measurements (*cf.* Figure 1d) are given in Table A1. The comparison of the fit to the experimental data is shown in Figure A2.

**Table A1:** Fitting parameters of the equivalent circuit shown in Figure 1d. The surface area of all the WEs was 0.5 cm${}^{2}$. $R_{1}$ and $R_{2}$ denote the resistance elements, C the capacitance element, CPE the constant phase element (Y for admittance (in $\Omega^{-1}$), and n the phase parameter (in A.U.)).

Sensor System/Treatment	$R_{1}$ [Ω]	$R_{2}$ [Ω]	*C* [F]	CPE (Y,n) [$\Omega^{-1}$; A.U.]
uncoated WE exposed to water	185	880	$3.47\times 10^{-10}$	$1.56\times 10^{-5}$; 0.699
EtOH-STF-coated WE exposed to water	187	4406	$5.08\times 10^{-8}$	$1.25\times 10^{-6}$; 0.765
THF-STF-coated WE exposed to water	186	3470	$3.47\times 10^{-8}$	$1.77\times 10^{-6}$; 0.779
THF-STF-coated WE exposed to ethanol	186	6270	$7.83\times 10^{-8}$	$3.61\times 10^{-6}$; 0.6528

## References

[1] Mohankumar P., Ajayan J., Mohanraj T., Yasodharan R. (2021). Recent developments in biosensors for healthcare and biomedical applications: A review. Measurement.

[2] Chen Y., Liu J., Yang Z., Wilkinson J.S., Zhou X. (2019). Optical biosensors based on refractometric sensing schemes: A review. Biosens. Bioelectron..

[3] Wang X.-D., Wolfbeis O.S. (2020). Fiber-optic chemical sensors and biosensors (2015–2019). Anal. Chem..

[4] Meng Z., Stolz R.M., Mendecki L., Mirica K.A. (2019). Electrically-transduced chemical sensors based on two-dimensional nanomaterials. Chem. Rev..

[5] Khan M.I., Mukherjee K., Shoukat R., Dong H. (2017). A review on pH sensitive materials for sensors and detection methods. Microsyst. Technol..

[6] Li W., Luo W., Li M., Chen L., Chen L., Guan H., Yu M. (2021). The impact of recent developments in electrochemical POC sensor for blood sugar care. Front. Chem..

[7] Cole L.A., Sutton-Riley J.M., Khanlian S.A., Borkovskaya M., Rayburn B.B., Rayburn W.F. (2005). Sensitivity of over-the-counter pregnancy tests: Comparison of utility and marketing messages. J. Am. Pharm. Assoc..

[8] Corman V.M., Haage V.C., Bleicker T., Schmidt M.L., Mühlemann B., Zuchowski M., Jo W.K., Tscheak P., Möncke-Buchner E., Müller M.A. (2021). Comparison of seven commercial SARS-Cov-2 rapid point-of-care antigen tests: A single-centre laboratory evaluation study. Lancet Microbe.

[9] Peeling R.W., Wedderburn C.J., Garcia P.J., Boeras D., Fongwen N., Nkengasong J., Sall A., Tanuri A., Heymann D.L. (2020). Serology testing in the COVID-19 pandemic response. Lancet Infect. Dis..

[10] Vandenberg O., Martiny D., Rochas O., van Belkum A., Kozlakidis Z. (2021). Considerations for diagnostic COVID-19 tests. Nat. Rev. Microbiol..

[11] Masson J.-F. (2017). Surface plasmon resonance clinical biosensors for medical diagnostics. ACS Sens..

[12] Magar H.S., Hassan R.Y.A., Mulchandani A. (2021). Electrochemical impedance spectroscopy (EIS): Principles, construction, and biosensing applications. Sensors.

[13] Killner M.H.M., Danieli E., Casanova F., Rohwedder J.J.R., Blümich B. (2017). Mobile compact 1H NMR spectrometer promises fast quality control of diesel fuel. Fuel.

[14] van Kollenburg G.H., van Manen H.-J., Admiraal N., Gerretzen J., Jansen J.J. (2021). Low-cost handheld NIR spectroscopy for identification of organic solvents and low-level quantification of water contamination. Talanta.

[15] Majchrzak T., Wojnowski W., Dymerski T., Gębicki J., Namieśnik J. (2018). Electronic noses in classification and quality control of edible oils: A review. Food Chem..

[16] Vempatapu B.P., Kanaujia P.K. (2017). Monitoring petroleum fuel adulteration: A review of analytical methods. Trends Anal. Chem..

[17] Wang B., Wu C., Reniers G., Huang L., Kang L., Zhang L. (2018). The future of hazardous chemical safety in china: Opportunities, problems, challenges and tasks. Sci. Total Environ..

[18] Athar M., Mohd Shariff A., Buang A., Shuaib Shaikh M., Ishaq Khan M. (2019). Review of process industry accidents analysis towards safety system improvement and sustainable process design. Chem. Eng. Technol..

[19] Henretig F.M., Kirk M.A., McKay C.A. (2019). Hazardous chemical emergencies and poisonings. N. Engl. J. Med..

[20] Kriaa S., Pietre-Cambacedes L., Bouissou M., Halgand Y. (2015). A survey of approaches combining safety and security for industrial control systems. Reliab. Eng. Syst. Saf..

[21] Şennik E., Çolak Z., Kılınç N., Öztürk Z.Z. (2010). Synthesis of highly-ordered TiO_2_ nanotubes for a hydrogen sensor. Int. J. Hydrog. Energy.

[22] Gebicki J. (2016). Application of electrochemical sensors and sensor matrixes for measurement of odorous chemical compounds. Trends Anal. Chem..

[23] Liu Z., Zhang S., Jin Y.M., Ouyang H., Zou Y., Wang X.X., Xie L.X., Li Z. (2017). Flexible piezoelectric nanogenerator in wearable self-powered active sensor for respiration and healthcare monitoring. Semicond. Sci. Technol..

[24] Kiew L.-V., Chang C.-Y., Huang S.-Y., Wang P.-W., Heh C.-H., Liu C.-T., Cheng C.-H., Lu Y.-X., Chen Y.-C., Huang Y.-X. (2021). Development of flexible electrochemical impedance spectroscopy-based biosensing platform for rapid screening of SARS-Cov-2 inhibitors. Biosens. Bioelectron..

[25] Wu X., Lu C., Han Y., Zhou Z., Yuan G., Zhang X. (2016). Cellulose nanowhisker modulated 3D hierarchical conductive structure of carbon black/natural rubber nanocomposites for liquid and strain sensing application. Compos. Sci. Technol..

[26] Slobodian P., Riha P., Lengalova A., Svoboda P., Saha P. (2011). Multi-wall carbon nanotube networks as potential resistive gas sensors for organic vapor detection. Carbon.

[27] Knopfmacher O., Hammock M.L., Appleton A.L., Schwartz G., Mei J., Lei T., Pei J., Bao Z. (2014). Highly stable organic polymer field-effect transistor sensor for selective detection in the marine environment. Nat. Commun..

[28] Lee M.Y., Kim H.J., Jung G.Y., Han A.-R., Kwak S.K., Kim B.J., Oh J.H. (2015). Highly sensitive and selective liquid-phase sensors based on a solvent-resistant organic-transistor platform. Adv. Mater..

[29] Rozman M., Štukovnik Z., Sušnik A., Pakseresht A., Hočevar M., Drobne D., Bren U. (2022). A HepG2 cell-based biosensor that uses stainless steel electrodes for hepatotoxin detection. Biosensors.

[30] Štukovnik Z., Bren U., Rozman M. (2021). Model electrochemical biosensor for the detection of methanol in aqueous solutions with yeast cells. Acta Chim. Slov..

[31] Bondarenko A.S., Ragoisha G.A., Pomerantsev A.L. (2005). Progress in Chemometrics Research.

[32] Dielectric Conestant of Common Solvents. https://depts.washington.edu/eooptic/linkfiles/dielectric_chart%5B1%5D.pdf.

